# A checklist for translating and adapting questionnaires (CTAQ) in healthcare research: insights from a Delphi method approach

**DOI:** 10.1186/s41182-025-00798-2

**Published:** 2025-11-07

**Authors:** Nguyen Tran Minh Duc, Kadek Agus Surya Dila, Duc Hoang Nguyen, Sameh Eltaybani, Amit G. Singal, Amna Rehana Siddiqui, Elisabeth Piault-Louis, Evangelos C. Fradelos, Farrukh Ansar, Filippo Maselli, Hyemin Han, Jeffery Hill, Juntra Karbwang, Latika Gupta, Martin L. Verra, Mohammad Karamouzian, Rama Chandran Nair, Shaw Bronner, Tara Ballav Adhikari, Ulrich S. Tran, Ulrik Havshøj, Darren Hedley, Delesha M. Carpenter, Filipa Alves da Costa, Francesca Esposito, K. Rivet Amico, Matthew DF McInnes, Nasia Safdar, Gladson Vaghela, Nguyen Tien Huy

**Affiliations:** 1 Molecular Neuroscience Research Center, Shiga University of Medical Science, Shiga, Japan; 2 Giri Emas Hospital, Buleleng, Singaraja City, Bali 81171 Indonesia; 3 Hanoi Medical University, Hanoi, Vietnam; 4 Cardiovascular Laboratory, Methodist Hospital, Merrillville, IN USA; 5 Global Nursing Research Center, The University of Tokyo, Tokyo, Japan; 6 UT Southwestern Medical Center, Dallas, TX USA; 7 Jinnah Sindh Medical University, Karachi, Pakistan; 8 Genentech, Inc, South San Francisco, CA USA; 9 Department of Nursing, University of Thessaly, Larissa, Greece; 10 Alkhidmat Raazi Hospital, Rawalpindi, Pakistan; 11 Department of Human Neuroscience -Sapienza, University of Rome, Rome, Italy; 12 Educational Psychology Program, University of Alabama, Tuscaloosa, AL USA; 13 Department of Emergency Medicine, University of Cincinnati, Cincinnati, OH USA; 14 Thammasat University, Bangkok, Thailand; 15 Department of Rheumatology, Royal Wolverhampton Hospitals NHS Trust, Wolverhampton, UK; 16 Francis Crick Institute, London, UK; 17 Department of Physiotherapy, Inselspital, Bern University Hospital, University of Bern, Bern, Switzerland; 18 Centre On Drug Policy Evaluation, MAP Centre for Urban Health Solutions, St. Michael’s Hospital, Toronto, ON Canada; 19 Dalla Lana School of Public Health, University of Toronto, Toronto, ON Canada; 20 University of Ottawa, Ottawa, Canada; 21 ADAM Center, Brooklyn, NY USA; 22 Department of Public Health, Aarhus University, Aarhus, Denmark; 23 Department of Cognition, Emotion, and Methods in Psychology, Faculty of Psychology, University of Vienna, Vienna, Austria; 24 Department of Anaesthesiology, Odense University Hospital, Odense, Denmark; 25 Olga Tennison Autism Research Centre, School of Psychology and Public Health, La Trobe University, Melbourne, VIC Australia; 26 Division of Pharmaceutical Outcomes and Policy, University of North Carolina Eshelman School of Pharmacy, Asheville, NC USA; 27 Research Institute for Medicines (iMED.ULisboa), Faculty of Pharmacy, University of Lisbon, Lisbon, Portugal; 28 Department of Psychology “Renzo Canestrari”, University of Bologna, Cesena, Italy; 29 Instituto de Ciências Sociais da Universidade de Lisboa, Lisbon, Portugal; 30 Department of Health Behavior and Health Equity, University of Michigan School of Public Health, Ann Arbor, MI USA; 31 Department of Radiology, University of Ottawa, Ottawa, ON Canada; 32 Department of Medicine, University of Wisconsin-Madison, Madison, WI USA; 33 Nuffield Department of Population Health, University of Oxford, Oxfordshire, UK; 34 Institute of Research and Development, Duy Tan University, Da Nang, Vietnam; 35 School of Medicine and Pharmacy, Duy Tan University, Da Nang, Vietnam; 36 School of Tropical Medicine and Global Health, Nagasaki University, Nagasaki, Japan; 37 WHO Collaborating Center for HIV Surveillance, Institute for Futures Studies in Health, Kerman University of Medical Sciences, Kerman, Iran; 38 School of Infection, Inflammation and Immunology, College of Medicine and Health, University of Birmingham, Birmingham, UK; 39 Oxford India Centre for Sustainable Development, University of Oxford, Oxford, UK

**Keywords:** Checklist, Delphi technique, Cross-cultural comparison

## Abstract

**Purpose:**

Accurate translation and adaptation of survey questionnaires are essential for ensuring validity and reliability in cross-cultural healthcare research. Despite the global expansion of healthcare studies, standardized guidelines for the translation process are limited.

**Methods:**

To address this gap, we developed the Checklist for Translating and Adapting Questionnaires (CTAQ). A three-round Delphi survey was conducted to refine and validate the CTAQ. An international panel of experts in survey methodology, cross-cultural research, and healthcare participated in the study, providing iterative feedback to achieve consensus on checklist items. The development of the CTAQ involved: (i) drafting an initial checklist based on a comprehensive literature review and expert insights; (ii) rating the importance and relevance of each item using an 80% consensus threshold; and (iii) revising items through successive Delphi rounds until consensus was reached.

**Results:**

The finalized CTAQ comprises eight stages: defining the target audience and objectives; forming a translation team; forward and backward translation; comparing versions; reconciliation; pretesting and evaluation; final review and proofreading; and post-survey evaluation. This structured approach, informed by expert consensus, integrates best practices and addresses cultural nuances, thereby enhancing the accuracy and reliability of translated survey instruments.

**Conclusions:**

The CTAQ offers a systematic, consensus-based framework that enhances the linguistic and cultural accuracy of translated survey instruments in healthcare research.

**Practice implications:**

Adopting the CTAQ standardizes translation workflows and promotes the production of valid, reliable, and culturally appropriate questionnaires. This contributes to greater rigor and quality in international and cross-cultural healthcare studies.

**Supplementary Information:**

The online version contains supplementary material available at 10.1186/s41182-025-00798-2.

## Introduction

Although questionnaires are widely used to gather information from study participants, health questionnaires are typically translated and used in diverse cultural and linguistic contexts after testing for content validity, construct validity, and reliability in an original language and context [1, 2]. For studies that span more than one country or culture, it is crucial to meticulously translate questionnaires into many languages to guarantee the study’s validity and reliability [3]. The process of translating a questionnaire has been outlined in several established works, including (i) the need for a translation team; (ii) the identification of the target audience and objectives; (iii) the actual translation process itself, sometimes called forward translation [4], and (iv) the final proofreading and validation [3, 5–7]. For better translation quality overall, there are a few optional steps to consider. One is back translation, which involves translating the original questionnaire into another language. Then, to avoid any non-equivalence or context-specific details, this translated version can be translated back into the original language [1, 6–8]. With the rapid global expansion of health research, the demand for accurately translated questionnaires has significantly increased.

The reliability, validity, and consistency of health questionnaire results across languages have been the subject of extensive research in their translation and culture [1, 5–10]. Tsang [11] and Ware [4] have emphasized the need for accurate measurement of pain medicine and quality of life (QoL) research. A reference validation study for the Quality of Life in Hand Eczema Questionnaire (QOLHEQ) was presented in a guide for translation and national validation of the QOLHEQ [12]. This study opens the possibility of harmonizing the national validation of the various language versions of the QOLHEQ, and the concept can be adapted to other contexts as needed. Another helpful reference is the FACIT translation methodology, which has been widely used for approximately 30 years [6]. Published ‘guidelines’ by researchers have evaluated scale structure, validity, responsiveness, repeatability, and interpretability including cross-cultural adaptability [9, 11–14].

Nevertheless, the majority of the current literature is based on individual studies or experts ’ opinions. Existing guidelines, such as the FACIT translation methodology and the guide for translation and national validation of the QOLHEQ, have made valuable contributions. However, these approaches are often context-specific and may not be universally applicable across all health domains. Moreover, while current guidelines evaluate scale structure, validity, responsiveness, repeatability, and interpretability, they often fall short in addressing the nuanced challenges of cultural adaptation beyond linguistic translation. Despite the importance of this issue, no multidisciplinary expert consensus has been established. To address this gap, we initiated the first Delphi consensus aimed at developing a systematic method for translating and validating research survey questionnaires. By outlining a consistent method for translating and validating these tools, this study will improve the overall quality of cross-cultural health research and provide researchers with a starting point for their investigations.

## Methods

### Rationale for using Delphi technique

A Delphi study was conducted from October 2023 to May 2024 to develop a comprehensive checklist for translating survey questionnaires in healthcare research. The Delphi method is widely accepted for achieving consensus among a panel of experts through a series of structured surveys [15]. Following the Conducting and REporting of DElphi Studies (CREDES) guidelines [16], the checklist development process is divided into five stages: (i) planning, (ii) initial checklist drafting, (iii) Delphi rounds for consensus building, (iv) checklist dissemination, and (v) checklist maintenance.

### Objectives

The objective of this study was to develop and validate the Checklist for Translating and Adapting Questionnaires (CTAQ), a practical tool designed to enhance the rigor of cross-cultural survey translation in healthcare research.

### Study design

A modified Delphi approach was used, following CREDES guidelines. The checklist development was structured into five stages: planning, drafting, Delphi rounds, dissemination, and maintenance.

### Participant selection and recruitment

A seven-member workgroup from four countries was formed to develop the CTAQ. Expert selection criteria included publication records in relevant fields, multidisciplinary expertise, and geographic diversity (Supplement 1). Experts were identified using Google Scholar (2012–2023) and invited via email.

### Informational input to panelists

Panelists received background on the study’s aims, draft checklist items, and procedural instructions prior to Round 1. Supporting literature and rationale for item selection were also provided.

### Prevention of bias

Panelist identities were blinded to each other. Responses were collected anonymously via SurveyMonkey. Qualitative and quantitative data were analyzed independently by the study team. Weekly reminders ensured balanced participation across regions.

### Delphi procedure and definition of consensus

A three-round Delphi process was conducted using SurveyMonkey (SurveyMonkey Inc., San Mateo, CA, USA; www.surveymonkey.com). Items were rated using a 5-point Likert scale (1 = strongly disagree to 5 = strongly agree). Consensus was defined a priori as a mean score of ≥ 4. Items below the threshold were revised or removed based on participant feedback.

### Stopping criteria

The process concluded after Round 3 when all remaining items reached consensus and further modifications were deemed unnecessary.

### Data analysis

Quantitative data were summarized using mean and standard deviation. Qualitative comments were thematically analyzed to inform item revisions.

### Checklist development and refinement

Checklist items were drafted through literature review and EQUATOR Network database screening. Draft items were independently reviewed by three pairs of researchers.

### Ethical considerations

As no personal data were collected, IRB approval was not required. Participation was voluntary, and all expert panelists consented via email.

### CREDES compliance

The CREDES checklist is included as Supplement 2 and maps each reporting requirement to the corresponding section in this manuscript.

## Results

The Delphi process comprised three rounds. In Round 1, all proposed items were rated and qualitative feedback collected. In Round 2, revised items were re-evaluated, and in Round 3, remaining discrepancies were resolved, resulting in final consensus across all items. From an initial pool of 563 identified experts, 30 consented to participate in the Delphi rounds. A final workgroup panel of 24 experts was established. The panel's expertise encompassed various disciplines, including public health, nursing research, medical and clinical sciences, physiotherapy, psychology, cognitive and emotional research, anaesthesiology, rheumatology, immunology, and pharmaceutical sciences.

From the first round of the Delphi process, we obtained response rates for each item and modified them accordingly, as described in Supplement 3. We used mean and standard deviation (SD) to present the summary ratings, with items having a mean of 4.0 or higher considered as reaching consensus. Items not reaching consensus were discussed; they were either modified for the next round or removed. In the first round of Delphi, one item (item 1.5) was removed due to low consensus. Another item (item 8.2) reached a low consensus score; however, because we considered this item essential, we modified it for the second round of Delphi. Detailed item-by-item changes across Delphi rounds, including removals, revisions, and modifications, are documented in Supplement 3 and Supplement 4.

Fourteen items (items 1.1, 1.4, 2.2, 3.4, 4.2, 4.3, 5.1, 5.2, 5.3, 6.4, 7.1, 7.3, 8.1, 8.3) were kept without modification. One item was removed (item 1.5), and seven items were removed and merged with other items or had descriptions added to the checklist (items 2.4, 2.5, 2.6, 2.7, 2.8, 2.9, 2.10). Fourteen items (items 1.2, 1.3, 2.1, 2.3, 3.1, 3.2, 3.3, 3.5, 4.1, 6.1, 6.2, 6.3, 7.2, 8.4) were revised and modified based on comments from the experts (Supplement 3). After the checklist was modified, it underwent a second round of evaluation by the experts.

From the second round of the Delphi process, we obtained response rates for each item and modified them accordingly, as described in Supplement 4. Two items (items 1.3 and 8.2) were removed based on experts ’ comments and low consensus, respectively. One item (item 4.3) reached a low consensus; however, because we considered this item essential, we modified it for the third round of Delphi.

Twenty items (items 1.1, 1.2, 1.4, 2.1, 2.2, 2.3, 3.1, 3.2, 3.3, 5.1, 5.3, 6.1, 6.2, 6.3, 6.4, 7.1, 7.2, 7.3, 8.3, 8.4) were kept without modification. Due to the removal of item 8.2, item 8.3 became item 8.2, and item 8.4 became item 8.3. Five items (items 3.5, 4.1, 4.2, 5.2, 8.1) were revised and modified based on comments from the experts (Supplement 4). After the checklist was modified , it underwent the third round of evaluation by the experts before finalization.

**Fig. 1:**
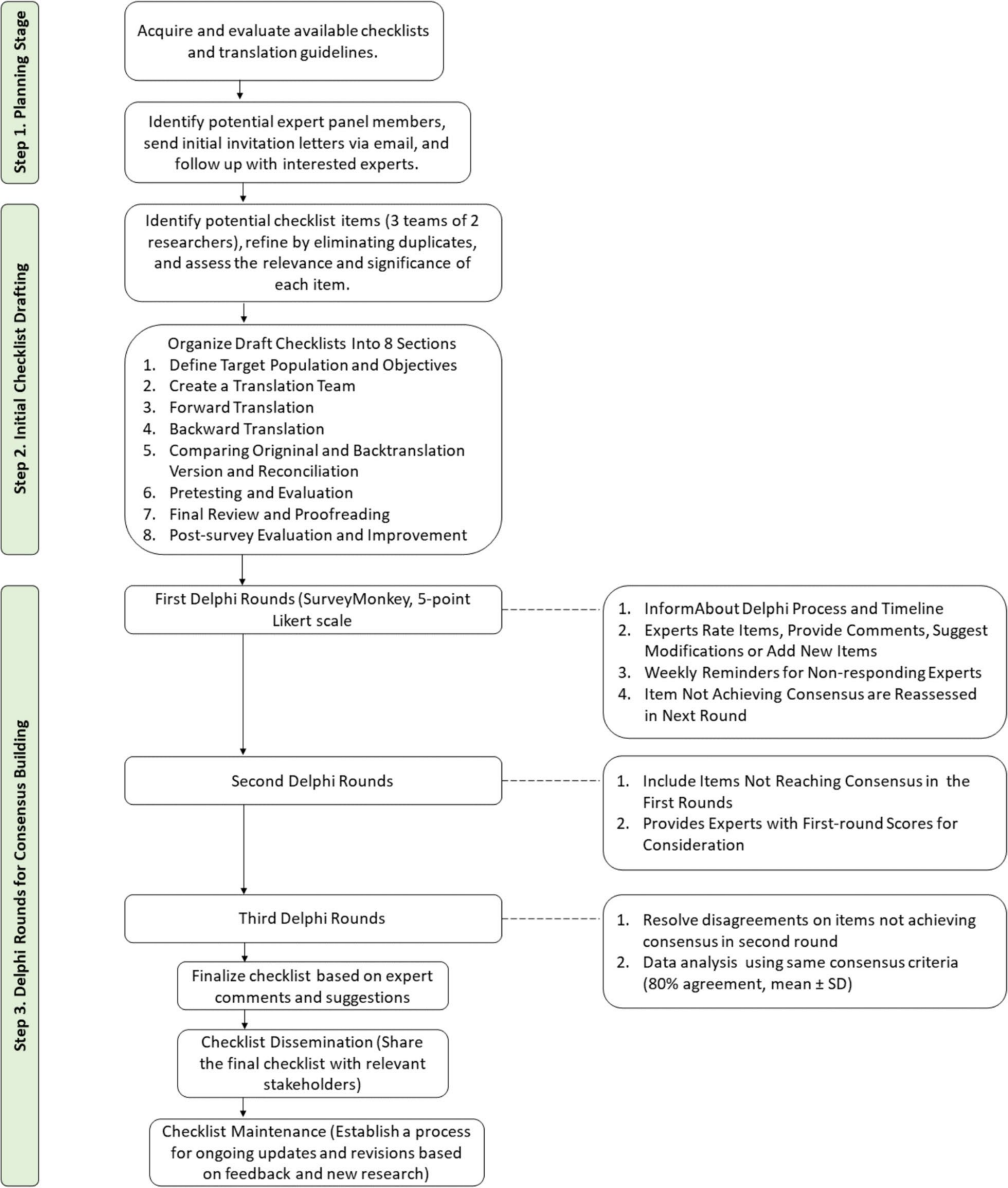
Process for developing and refining the initial checklist following CREDES guideline

After the third round, the final checklist items are presented in Table 1. The final items were categorized into eight domains. The finalized CTAQ checklist included 27 items (Fig. 1; Table 1), each representing a critical step in the translation and adaptation of questionnaires. All items achieved an average rating of 4.0 or higher in the final round. Most of the qualitative feedback from the experts in this round was related to the wording of the checklist rather than its content.

**Table 1:** Percentage agreement and mean score with standard deviation of the items in third rounds of Delphi (final rounds)

Item	Item description	Round 3 agreement mean (SD)
Define target audience and objectives		
1.1	Communicate the objective before the translation process, including the study objective, target respondent, and translation objective	4.42 (0.96)
1.2	Specify the target respondent or the target population's characteristics, including: - Nationality and regional differences - Language dialects spoken within the community - Cultural background and perspectives relevant to the subject matter - Educational levels of the target population - Socioeconomic status	4.21 (0.63)
1.3	Engage with a diverse group of stakeholders, such as interviewers, pretest respondents, and experts in the field, to collect input and perspectives	4.26 (0.93)
Create a translation team		
2.1	Form a translation team with a translation lead or manager, at least two proficient translators, preferably native speakers of the target language, and, if applicable, the source language questionnaire developer	4.32 (0.67)
2.2	Appoint a qualified translation manager: - Identify an experienced individual who will oversee the entire translation process (manage resources, direct and coordinate procedural steps, documentation of results, and facilitate discussion) - Verify that the translation manager has a strong understanding of the survey’s objectives and the target audience	4.26 (0.81)
2.3	Define the translation team’s roles and responsibilities: - Outline the distinct roles of each team member, including a research team representative, translators, linguistic experts, healthcare professionals, and, if applicable, the source-language questionnaire developer	4.33 (0.59)
Forward translation		
3.1	Select translators: - A minimum of two translators fluent in the source language, native/near-native speakers of the target language - Expertise/experience in translating in the subject matter field (If applicable) - Familiar with the culture and context of the target audience	4.53 (0.51)
3.2	Independent translation: - Each translator works independently on translating the questionnaire	4.42 (0.69)
3.3	Identify discrepancies: - Identify who will compare the translations and identify discrepancies, e.g., researchers or an independent translator - Resolve discrepancies through discussion and consensus between the translators	4.68 (0.48)
3.4	Prioritize understandability: - Use simple and clear language in the translated questionnaire (avoid medical jargon or technical terms) - Maintain consistency in terminology throughout the questionnaire - Be culturally sensitive; account for conceptual differences - Retain original tone and intent	4.58 (0.51)
3.5	Capture conceptual equivalents: - Aim for conceptual equivalence over word-for-word translation or etymological equivalence - Culturally appropriate for the audience	4.58 (0.69)
Back translation		
4.1	Choose back translators: - Formulate well-defined criteria for translators and report them in publication - Formulate well-defined objectives of translation and (potential) target audience - Two independent back translators are recommended to minimize the risk of bias. Any conflict should be addressed by agreement or by a third (senior) reviewer - Back translators should not have access to the questionnaire in its original language	4.53 (0.51)
4.2	Perform independent back translation: - Parallel comparison of forward and backward versions - Prefer flexible and readable translation over word-for-word	4.42 (0.69)
4.3	Implement double-back-translation methodology (optional) - Have two independent expert translators translate the source material into the target language - Involve a third impartial translator to resolve disputes between two translators and consolidate a final version - Blindly back-translate the reconciled version into the source language - Check the back-translation for compatibility and equivalence with the source version - Involve multiple independent reviewers to choose the best translation for each item or suggest alternatives - Seek consensus among the organizing team and language coordinator - Test the target-language version with native-country patients and incorporate their feedback for revisions	4.05 (0.85)
Comparing original and back-translated versions and reconciliation		
5.1	Side-by-side comparison: - Compare the original questionnaire with the back-translated version side by side - Identify any discrepancies or ambiguities in concept or wording between the two versions	4.58 (0.69)
5.2	Resolution of discrepancies: - Engage in discussions with the translation team to address identified discrepancies - Strive for consensus to create a harmonized version that retains the original meaning - A high level of inconsistency may result in restarting the process from step 3 (Forward translation)	4.58 (0.61)
5.3	Involvement of translation manager: - The Translation Manager should lead discussions with both forward and backward translators - Make necessary changes to the back-translation based on input and consensus - Be prepared for multiple iterations if required until the translation team is satisfied with the harmonized version	4.32 (0.67)
Pretesting and evaluation		
6.1	Pretest with target audience: - Conduct pretesting of the translated questionnaire with members of the target audience - Assess clarity, comprehension, and cultural appropriateness - Conduct cognitive interviews to uncover issues - Debrief for open-ended feedback on full experience	4.61 (0.61)
6.2	Representative pretest group: - Recruit from geographic areas where the target population resides - Ensure demographic diversity by including individuals of varying ages, genders, ethnicities, and educational backgrounds in the pretesting process as appropriate	4.42 (0.69)
6.3	Consider sample size: - Refer to research on recommended sample sizes for cognitive interviews - Start with a small sample of participants for initial pretesting - Calculate the sample based on the estimated saturation point to uncover issues	4.32 (0.67)
6.4	Revisions based on feedback: - Incorporate necessary revisions to the questionnaire based on pretest feedback - Improve clarity and comprehension of questions and instructions - Fix any content or grammar issues raised	4.47 (0.51)
Final review and proofreading		
7.1	Expert review: - Have subject-matter experts or researchers experienced in survey methodology review the translated questionnaire - Assess content validity, cultural appropriateness, and clarity through expert feedback	4.63 (0.50)
7.2	Final validation: - Assess internal consistency and reliability using appropriate indices such as Cronbach's alpha, McDonald's omega, or other relevant reliability measures - Use Cronbach’s alpha to measure the internal consistency and reliability of survey items - Use measures such as the Kappa coefficient and intraclass correlation coefficient to assess item-to-item agreement and equivalence between the source and target instruments	4.33 (0.77)
7.3	Proofreading: - Conduct a meticulous proofreading of the final translated questionnaire - Check for language accuracy, formatting consistency, and overall quality - Ensure the questionnaire is free of typos, grammatical errors, and formatting inconsistencies	4.67 (0.49)
Post-survey evaluation and continuous improvement		
8.1	Conduct post-survey evaluations: - A conventional and readily accessible (preferably online) survey - Feedback should be collected from both translators and readers - (Optional) Put a QR code in the survey in case participants have any feedback	3.84 (0.69)
8.2	Perform real-world revisions: - Use the feedback from the post-survey evaluations to revise the translated instrument - Address any clarity, comprehension, or cultural sensitivity issues identified during the survey administration	4.16 (0.76)
8.3	Document revisions: - Maintain clear documentation of all revisions made for future reference and quality control	4.56 (0.51)

## Discussion

Translating questionnaires is essential for conducting survey research across populations with different languages. While English is often used internationally, adapting questionnaires to the target population’s language offers advantages such as improved clarity, reduced non-response rates, and increased accessibility for respondents with varied educational backgrounds. Our Delphi study identified eight key steps for translating questionnaires: (1) define target audience and objectives, (2) create a translation team, (3) perform forward and backward translation, (4) compare original and back-translated versions, (5) reconcile differences, (6) pretest and evaluate, (7) conduct final review and proofreading, and (8) implement post-survey evaluation and continuous improvement.

### Defining target audience and setting objective

The first steps in the translation process are determining the target audience and the survey’s objectives. Survey questions must be relevant and understandable, which requires understanding the target population’s linguistic preferences and cultural nuances. Unlike previous studies, which did not clearly state the importance of setting objectives other than translation objectives, [10, 17, 18] our study recommends discussing and determining study objectives at the beginning of the translation procedure. By setting clear objectives, researchers can ensure that the translated questionnaire accurately captures the intended constructs and make meaningful data collection more accessible. With the potential variability in researchers’ and translators’ perspectives [7, 19], the team must agree on the study objectives before the commencement of the translation.

The question objectives, or aims, are crucial in translation. Translators must fully understand the intended meaning of each question in the original language. The core research team should clarify these objectives to the translation team, as subtle nuances may not be immediately apparent and could be interpreted differently. Misunderstandings at this stage could significantly impact the accuracy of translations and, consequently, the distribution of responses. Clear communication between the research and translation teams is essential to ensure that the true intent of each question is preserved [7].

Translator selection should align with the target respondents’ backgrounds, favoring multilingual individuals who share their national, regional, educational, and cultural perspectives to ensure accessibility, especially for those with limited formal education. A conceptual rather than literal translation is essential, as direct translations often produce awkward phrasing and fail to reflect linguistic variations, politeness norms, and cultural nuances. Instead, conceptual translation preserves the intended meaning by considering the respondents’ knowledge, beliefs, and values, ensuring the questionnaire remains both relevant and comprehensible [7, 20].

### Creating a translation team

Accurate and culturally relevant translations require the participation of a varied and skilled translation team. Researchers should assemble a team that is fluent in both the source and target languages. Furthermore, adding people with subject matter and cultural expertise can improve the compatibility of the translated questionnaire. Based on our Delphi study, we propose that a translation team should at least consist of (1) a translation lead or manager; (2) a minimum of two translators who are proficient in both source and target languages, (preferably native speakers of the target language to capture nuances or cultural perspectives); (3) a multidisciplinary team to review translation results that consist of a research study member, a linguistic expert on the target language, a healthcare professional with specific competence in the source and target languages, as well as the topic of the questionnaire, and (4) invite the source language questionnaire developer if using a pre-existing questionnaire.

Our proposed translator team composition aligns closely with that described by Beaton et al. [9] However, we have introduced an additional crucial role not previously mentioned: the translation lead or manager. This addition is supported by the IMIA guide, which recommends establishing central translation management [21]. A designated individual should be appointed to manage and coordinate translation tasks. This person is responsible for assessing translation needs, ensuring proper adherence to the translation process, and facilitating discussions among team members. The inclusion of this role enhances the overall efficiency and quality of the translation process.

While previous studies and guidelines noted the importance of linguistic proficiency [7, 21], our guide emphasizes the inclusion of native speaker translators and healthcare professionals with specific competence in the source and target language as well as the topic of the questionnaire to enhance the translation’s relevance and accuracy and to complement if a linguistic expert is not available for the target language. Also, the inclusion of source language questionnaire developer (when using a pre-existing questionnaire) can provide valuable insights into the intended meaning of items.

### Forward and backward translation

Forward and backward translations are essential for verifying the translation's accuracy and consistency. Linguistic equivalency is ensured by translating the questionnaire from the source language to the target language via forward translation. In order to identify inconsistencies and ambiguities, backward translation entails having separate translators translate the questionnaire back into its original language. This iterative procedure reduces the possibility of misunderstanding and improves the translation.

Forward translation is the first step of the questionnaire translation process. Based on our Delphi round and experts’ input, a minimum of two independent bilingual forward translators who are native speakers of the target language and have high proficiency in the source language is required. The results were then compared, and any discrepancies related to wording choice between the translators were noted, discussed, and resolved. Our study aligns with guidelines described by Beaton et al. [9] that point to the importance of using a bilingual translator with target language as their mother tongue. Additionally, we also recommend aiming for conceptual equivalence in translation and maintaining understandability by using simpler and clear language, creating a glossary of key terms, maintaining consistency in terminology, original tone and intent, as well as being culturally sensitive. In addition, Beaton et al. also recommended that the two forward translators should have different profiles or backgrounds, one that is aware of the concepts being examined in the questionnaire being translated and one that is not aware of it (a naïve translator) to achieve adequacy and equivalence as described by Povoroznyuk et al. [17].

The IMIA guide [21] does not recommend a back-translation procedure because adaptations made by the forward translators, which perfectly convey meaning from the original versions of the questionnaire, could be lost in the process of back translation. However, based on our Delphi study, back translation remains valuable, and thus, was included in our guide. It helps to identify discrepancies while still ensuring the translated questionnaire maintains the original meaning. In other words, this process acts as quality control to ensure linguistic accuracy and cultural relevance. To overcome the problem which is mentioned in the IMIA guide, we recommend choosing a back translator who is proficient in the target language, involving a minimum of two back translators, who work independently with a parallel comparison procedure and do not have access to the original/source language questionnaire. Optionally, the double back-translation procedure initially introduced by Eremenco et al. could be applied to enhance the precision and cultural adjustments needed [6].

### Comparing original and back-translated versions

Researchers can evaluate the accuracy of the translation by contrasting the back-translated version of the questionnaire with the original one. Differences between the two versions might point to unclear or culturally insensitive areas that need more work. Researchers can ensure the integrity of the survey instrument by identifying and addressing potential translation problems through a comprehensive comparison.

Based on the current experts’ input, a side-by-side comparison between original and back-translated versions is recommended. This step is important as a validity check to ensure consistency of translation across versions or target language and maintain the meaning of the original questionnaire [9]. Close attention should be given to each back-translated item, when compared to the original version, and any differences or inconsistencies across versions should be noted [17, 22].

### Reconciliation

In the reconciliation step, the translation team’s feedback is combined, and any differences found during the forward and backward translation processes are resolved. This cooperative effort makes sure that, while taking into consideration linguistic and cultural nuances, the final translated questionnaire faithfully captures the original instrument’s intended meaning. The translator lead or manager should lead and facilitate the discussion, aligning with a recommendation from the IMIA guide [21]. If discrepancies cannot be resolved, we recommend preparing an iterative translation process. Alternatively, maintaining more than one version with the target language-specific variations in the wording of questionnaire items could be an option [17]. Researchers can improve the translation’s clarity and relevance by conversing with translators and the linguistic experts in the team. In addition, similar to Beaton et al. [9], we emphasize that all issues and the rationale for each decision should be appropriately documented.

### Pretesting and evaluation

Our guide recommends a pretest procedure to assess the translated questionnaire’s comprehensibility, relevance, and cultural appropriateness with a representative sample of the target audience. It is conducted along with an evaluation to improve the quality of the survey instrument by identifying potential problems or misunderstandings through pretesting and making the required modifications. Through feedback collection from participants, researchers may verify the translation’s efficacy and make necessary, well-informed improvements. According to Beaton et al. [9], ideally 30–40 target samples should be tested. Each respondent should complete the questionnaire and undergo a cognitive interview as described by Meadows [23] to determine what the respondent thought about the meaning of each questionnaire item and each response provided. Both are then examined for different meanings perceived across the respondents for each item in the questionnaire and a high proportion of missing items or single response [9]. This process aims to reach saturation when no new or different meanings are perceived for each item, indicating that collecting additional data would not likely enhance understanding of the original intended meaning [17].

### Final review and proofreading

During the final review, the translated questionnaire is thoroughly proofread by language specialists to ensure correctness, consistency, and readability, thereby minimizing translation errors and enhancing overall quality.

Of equal importance is to ensure the cross-cultural validity of the translated questionnaire [17, 22], i.e., the current version of the questionnaire retains not just translation accuracy, but also the psychometric or measurement properties of the original version. The new questionnaire should preserve the concept validity, responsiveness, and reliability at the score level as well as item-level features, including internal consistency and item-to-scale correlations, as outlined by Beaton et al. [9]. Construct validity, reliability, and responsiveness are the three score-level properties that are fully assessed in the last phase. These test results are then contrasted with the results of similar tests conducted using the original questionnaire in the original setting. It is anticipated that the translated version would function similarly.

The final review and proofreading stage involves ensuring the accuracy, consistency, and clarity of the translated questionnaire. This process includes conducting a comprehensive assessment of the translated version's psychometric properties, such as construct validity, reliability, and responsiveness, to ensure that the translated instrument performs comparably to the original version in its intended setting. This assessment helps confirm that the translation maintains the original questionnaire’s integrity and provides reliable data across different cultural contexts.

### Post-survey evaluation and continuous improvement process

Assessing the success of the translation and pinpointing areas in need of development is crucial, even after the survey has been completed. Survey data analysis and respondent feedback requests can yield important information about the interpretability and applicability of the translated questionnaire. Furthermore, using a continual improvement process, researchers can apply the knowledge gained from the translation effort for other survey initiatives, thereby raising the caliber and significance of cross-cultural research studies.

### Limitations

We fully acknowledge the limitations inherent to the Delphi method and participant selection process. These include potential bias in expert selection and the possibility that the consensus-building approach may not capture the full diversity of opinions present in a larger, more varied panel. These factors should be considered when interpreting our results.

### Practice implications

The Checklist for Translating and Adapting Questionnaires (CTAQ) provides a practical, standardized roadmap for researchers and healthcare professionals involved in cross-cultural studies. By following its eight-stage framework, developed through expert consensus, the CTAQ enhances translation accuracy, cultural appropriateness, and methodological rigor. Recently, while generative AI translation tools can efficiently support initial drafts, their cultural and conceptual limitations require careful human oversight and systematic review [24, 25]. The CTAQ addresses these limitations by integrating rigorous expert consensus and iterative refinement, optimizing the accuracy and reliability of translations. This structured approach supports consistent, transparent practices across diverse research settings, enabling the development of valid and reliable survey instruments. Implementing the CTAQ can improve data quality, reduce measurement bias, and strengthen the credibility of findings in international and multilingual healthcare research.

## Conclusion

Translating survey questionnaires is a complex process that requires careful consideration of methodological, linguistic, and cultural contexts. Researchers can follow a systematic approach to ensure the validity, reliability, and cross-cultural compatibility of survey instruments. By extracting input using a Delphi method from experts in the field of survey research, this study provides a comprehensive step-by-step framework for translating survey questionnaires; therefore, promoting a thorough and meaningful instrument for cross-cultural survey research.

## Supplementary Information

Supplementary Material 1.

## Data Availability

The data supporting the findings of this study are available from the corresponding author upon reasonable request.

## References

[1] Rahman A, Iqbal Z, Waheed W, Hussain N. Translation and cultural adaptation of health questionnaires. J Pak Med Assoc. 2003;53(4):142–7. 12776898

[2] Hadier SG, Yinghai L, Long L, Hamdani SMZH, Khurram H, Hamdani SD, et al. Urdu translation and cross-cultural adaptation of canadian assessment of physical Literacy-2 (CAPL-2) questionnaires: a reliability analysis in Pakistani Children. New Dir Child Adolesc Dev. 2024;2024(1):9611010. 10.1155/2024/9611010.

[3] Chang AM, Chau JPC, Holroyd E. Translation of questionnaires and issues of equivalence. J Adv Nurs. 1999;29(2):316–22. 10.1046/j.1365-2648.1999.00891.x. 10197930 10.1046/j.1365-2648.1999.00891.x

[4] Ware JE, Keller SD, Gandek B, Brazier JE, Sullivan M. Evaluating translations of health status questionnaires methods from the IQOLA project. Int Qual Life Assess Int J Technol Assess Health Care. 1995;11(3):525–51. 10.1017/s0266462300008710 7591551

[5] Del Greco L, Walop W, Eastridge L. Questionnaire development: 3. Translation. CMAJ Can Med Assoc J. 1987;136(8):817–8. 3567792 PMC1492131

[6] Eremenco SL, Cella D, Arnold BJ. A comprehensive method for the translation and cross-cultural validation of health status questionnaires. Eval Health Prof. 2005;28(2):212–32. 15851774 10.1177/0163278705275342

[7] McKay RB, Breslow MJ, Sangster RL, Gabbard SM, Reynolds RW, Nakamoto JM, et al. Translating survey questionnaires: lessons learned. New Dir Eval. 1996;1996(70):93–104. 10.1002/ev.1037.

[8] Herdman M, Fox-Rushby J, Badia X. “Equivalence” and the translation and adaptation of health-related quality of life questionnaires. Qual Life Res. 1997;6(3):237–47. 9226981 10.1023/a:1026410721664

[9] Beaton DE, Bombardier C, Guillemin F, Ferraz MB. Guidelines for the process of cross-cultural adaptation of self-report measures. Spine. 2000;25(24):3186–91. 11124735 10.1097/00007632-200012150-00014

[10] Hall DA, Zaragoza Domingo S, Hamdache LZ, Manchaiah V, Thammaiah S, Evans C, et al. A good practice guide for translating and adapting hearing-related questionnaires for different languages and cultures. Int J Audiol. 2018;57(3):161–75. 29161914 10.1080/14992027.2017.1393565

[11] Tsang S, Royse CF, Terkawi AS. Guidelines for developing, translating, and validating a questionnaire in perioperative and pain medicine. Saudi J Anaesth. 2017;11(Suppl 1):S80–9. 28616007 10.4103/sja.SJA_203_17 PMC5463570

[12] Oosterhaven JAF, Schuttelaar MLA, Apfelbacher C, Diepgen TL, Ofenloch RF. Guideline for translation and national validation of the quality of life in hand eczema questionnaire (QOLHEQ). Contact Dermatitis. 2017;77(2):106–15. 28481015 10.1111/cod.12788

[13] Alavi M, Le Lagadec D, Cleary M. Challenges of cross-cultural validation of clinical assessment measures: a practical introduction. J Adv Nurs. 2025. 10.1111/jan.16906. 40129093 10.1111/jan.16906 PMC12721952

[14] Albabtain B, Paudyal V, Cheema E, Bawazeer G, Alqahtani A, Bahatheq A, et al. Translation, cultural adaptation and validation of Patient Satisfaction with Pharmacist Services Questionnaire (PSPSQ) 2.0 into the Arabic language among people with diabetes. PLoS ONE. 2024;19(6):e0298848. 10.1371/journal.pone.0298848. 38935668 10.1371/journal.pone.0298848 PMC11210780

[15] Okoli C, Pawlowski SD. The delphi method as a research tool: an example, design considerations and applications. Inf Manag. 2004;42(1):15–29.

[16] Jünger S, Payne SA, Brine J, Radbruch L, Brearley SG. Guidance on conducting and reporting DElphi studies (CREDES) in palliative care: recommendations based on a methodological systematic review. Palliat Med. 2017;31(8):684–706. 28190381 10.1177/0269216317690685

[17] Povoroznyuk R, Dzerovych N, Povoroznyuk V. A new voice: translating medical questionnaires. J World Lang. 2016;3(2):139–59.

[18] Kalfoss M. Translation and adaption of questionnaires: a nursing challenge. SAGE Open Nurs. 2019;5:2377960818816810. 33415214 10.1177/2377960818816810 PMC7774397

[19] Coskun Benlidayi I, Gupta L. Translation and cross-cultural adaptation: a critical step in multi-national survey studies. J Korean Med Sci. 2024;39(49):e336. 39716865 10.3346/jkms.2024.39.e336 PMC11666326

[20] Poot CC, Meijer E, Fokkema M, Chavannes NH, Osborne RH, Kayser L. Translation, cultural adaptation and validity assessment of the Dutch version of the eHealth literacy questionnaire: a mixed-method approach. BMC Public Health. 2023;23(1):1006. 10.1186/s12889-023-15869-4. 37254148 10.1186/s12889-023-15869-4 PMC10227819

[21] Txabarriaga R. IMIA guide on medical translation. Int Med Int Assoc. 2009;2:1–20.

[22] Lundanes E, Roten SM, Falkenberg HK, Leren L, Sundling V. Translation, cross-cultural adaptation, and validation of the Norwegian version of the Keratoconus outcomes research questionnaire. J Patient-Rep Outcomes. 2025;9(1):57. 10.1186/s41687-025-00896-z. 40402367 10.1186/s41687-025-00896-z PMC12098238

[23] Meadows K. Cognitive interviewing methodologies. Clin Nurs Res. 2021;30(4):375–9. 33998325 10.1177/10547738211014099

[24] Kasneci E, Sessler K, Küchemann S, Bannert M, Dementieva D, Fischer F, et al. ChatGPT for good? On opportunities and challenges of large language models for education. Learn Individ Differ. 2023;103:102274.

[25] Kung TH, Cheatham M, Medenilla A, Sillos C, Leon LD, Elepaño C, et al. Performance of chatGPT on USMLE: potential for AI-assisted medical education using large language models. PLoS Digit Health. 2023;2(2):e0000198. 10.1371/journal.pdig.0000198. 36812645 10.1371/journal.pdig.0000198 PMC9931230

