# Supplementary material for: A checklist for translating and adapting questionnaires (CTAQ) in healthcare research: insights from a Delphi method approach
Source: Trop Med Health. 2025 Nov 7;53:154. doi: 10.1186/s41182-025-00798-2 (PMC12593896; doi:10.1186/s41182-025-00798-2)
Supplement: Supplementary file 1 — Supplementary Material 1. [file 41182_2025_798_MOESM1_ESM.docx]

**Supplement 1: Expert Selection Criteria**

- Experts with a strong publication record (high-impact researchers with significant citation metrics) in survey development, translation, cross-cultural adaptation, and linguistic validation.(as first/second/last author, with at least 2 survey research publications, published in reputable journal or indexed in Scopus, PubMed, IF between 2-5, well cited or at least 5 citation within the past 10 years)
- Multidisciplinary representation to ensure comprehensive expertise.
- Geographic and cultural diversity (Experts from different nationality/country and if possible, from different region within the country) for varied perspectives.
- Commitment to active participation and consideration of diverse views in the Delphi process.

**Supplement 2: CREDES Checklist for Reporting Delphi Studies**

| **Item** | **Recommendation** | **Addressed in Manuscript** |
| --- | --- | --- |
| 1. Rationale for the choice of the Delphi technique | Justification of the choice of the Delphi technique as a method to address the research question. | Described in the "Methods" section, first paragraph, under the Methods heading. |
| 2. Planning and design | Description of how the method was designed to answer the research question. | Explained in the "Planning Stage" subsection of the Methods section. |
| 3. Definition of consensus | A priori definition of consensus. | Detailed in the "Delphi Rounds for Consensus Building" subsection, third paragraph of the Methods section. |
| 4. Informational input | Explanation of what information was provided to the participants before the first round. | Found in the "Initial Checklist Drafting" subsection of the Methods section. |
| 5. Prevention of bias | Description of measures taken to prevent bias. | Explained in the "Delphi Rounds for Consensus Building" subsection in the Methods section. |
| 6. Interpretation and processing of results | Explanation of how the results were processed and interpreted. | Covered across all subsections of the Results section. |
| 7. External validation | Report of any external validation. | Not applicable to this study. |
| 8. Purpose and rationale | Clear description of the purpose of the study. | Described in the last paragraph of the Introduction section. |
| 9. Expert panel | Explanation of how the expert panel was chosen. | Detailed in the "Planning Stage" subsection of the Methods section. |
| 10. Description of the methods | Detailed description of the methods used. | Presented in the entirety of the Methods section. |
| 11. Procedure | Step-by-step description of the Delphi process. | Described in the "Delphi Rounds for Consensus Building" subsection of the Methods section. |
| 12. Definition and attainment of consensus | Clear explanation of how consensus was defined and determined. | Detailed in the "Delphi Rounds for Consensus Building" subsection of the Methods section. |
| 13. Results | Reporting of results for each round separately. | Reported across all subsections of the Results section. |
| 14. Flow of participants | Flow diagram showing the number of participants involved in each round. | Described in the "Expert Panel Composition" subsection of the Results section and depicted in Figure 1. |
| 15. A list and description of participants | Characteristics of all participants. | Detailed in the "Expert Panel Composition" subsection of the Results section. |
| 16. Discussion of limitations | Discussion of the limitations of the study. | Detailed in the "Limitations" subsection of the Results section. |

**Supplement 3: Summary of Round 1 Delphi Survey Results with Expert Panel**

| **Item** | **Item description** | **Mean (SD)** | **Expert's comments** | **Action (keep/modify/removed)** |  |
| --- | --- | --- | --- | --- | --- |
|  |  |  |  |  |  |
| **Define Target Audience and Objectives** | | | | |  |
| 1.1 | Communicate the objective before the translation process, including the study objective, question objective, target respondent, and translation objective. | 4.33 (1.11) | (1) "Language is sensitive by the type of respondent therefore study objectives and target populations are necessary to be known" (2) "This would set the tone and allow for the rest of the project to be successful" (3) "Just include "the" before translation process" (4) "Included item "1.2". Item "1.2" is more comprehensive. So I recommend deleting item "1.1" and keeping item "1.2" (4) "Critical to precisely define" | Keep it to the next round |  |
| 1.2 | Determine target respondent or the target population's characteristics, including: | 4.13 (1.20) | (1) "Detailed characteristics not required" (2) "I am not sure the nationality is relevant here. I think the most important thing is the language spoken by the respondents." (3) "Not sure if the educational status applies here. Meaning some scales that are targeting healthcare staff asume that they have a level of education while other for the public or patients don't" | We modified it into new description as follows: Specify the target respondent or the target' population's characteristics including: - Nationality and regional differences - Language dialects spoken within the community - Cultural background and perspectives relevant to the subject matter - Educational levels of the target population - Socioeconomic status |  |
|  | - Nationality and regional differences |  |  |  |  |
|  | - Language dialects spoken within the community. |  |  |  |  |
|  | - Cultural background and perspectives relevant to the subject matter. |  |  |  |  |
|  | - Educational or literacy levels of the target population. |  |  |  |  |
| 1.3 | Evaluate the importance of conceptual translation to enhance the overall quality of the translated questionnaire. | 4.13 (1.20) | (1) "If it happens to be for a validated tool for scoring" (2) "This is crucial. A botched translation can destroy the entire project." (3) "Probably include some comparison, e.g., "Importance of conceptual translation (instead of verbatim translation)" (4) "It would be helpful to define "conceptual translation" here | We modified this item as follows: Evaluate the importance of conceptual translation (instead of verbatim translation) to enhance the overall quality of the translated questionnaire. |  |
| 1.4 | Engage with a diverse group of stakeholders, such as interviewers, pretest respondents, and experts in the field, to collect input and perspectives. | 4.39 (0.92) | (1) "I'd specify members of the target population specifically as folks who should be engaged" (2) "pilot testing need to suffice with the above before translation" (3) "While very important, this can be tricky. Selecting the appropriate stakeholders is very important in this process" (4) "Question is unclear and double barreled (what if I think experts and respondents are important but not I terviewers?); at what stage are you referring to? Translation? Before? After? All stages? | Keep it to the next round |  |
| 1.5 | Consider the social-economic status of the target audience, especially regarding their literacy level. If applicable, plan to use simple and clear language to ensure comprehension. | 3.96 ( 1.12) | (1) "This also affects preferred survey mode: paper, online, telephone" (2) "Especially if the tools self-administered, for interview administered training and manuals to be formulated" (3) "Isn't this is already covered in 1.2?" (4) "I suggest to deleting the reference to respondent's socio-economic status as may involve a class bias. Just focus on literacy levels and the use of simple and clear language" (5) "How does somenone determine that?" (6) "In some countries socio economic may not always be related so this would vary by region" (7) "SImple and clear language should be use all the times, wherever possible" (8) "Included in item 1.2" | Item removed |  |
| **Create a Translation Team** | |  |  |  |  |
| 2.1 | Form a translation team, consist of: | 4.26 (0.90) | (1) "Also include members of the target population"(2) "Study team need to be involved overall with a subgroup of translators"(3) "Both linguistic and health care competencies are essential for this step to be successful"(4) "Consisting of", "a minimum of 3 translators", currently the formulation is not clear regarding that linguistic expert and healthcare professional can be already part of 3-person team" | We modified this item as follows:Form a translation team with a Translation Lead or Manager, at least two proficient translators, preferably native speakers of the target language, and if applicable, the source-language Questionnaire Developer |  |
|  | - Translation Lead or Manager |  |  |  |  |
|  | - A minimal 3 Translator with linguistic competence in the source and target languages (2 Forward Translator, 1 Back Translator) |  |  |  |  |
|  | - Linguistic expert on target language |  |  |  |  |
|  | - A healthcare professional with specific competence in the source and target languages |  |  |  |  |
|  | - The source-language Questionnaire Developer (if possible). |  |  |  |  |
| 2.2 | Appoint a qualified Translation Manager: | 4.30 (1.04) | (1) "Sometimes it is a challenge and translation support team fills the gap" (2) "1.Not all research teams have these resources or funds to create such roles? 2.How is this different than a typical research coordinator?" | Keep it to the next round |  |
|  | - Identify an experienced individual who will oversee the entire translation process (manage resources, direct and coordinate procedural steps, documentation of results and facilitate discussion). |  |  |  |  |
|  | - Verify that the Translation Manager has a strong understanding of the survey's objectives and the target audience. |  |  |  |  |
| 2.3 | Define the Translation Team's Roles and Responsibilities: | 4.48 (0.71) | "Usually the translator teams comprise of study teams coupled with external translator" | We modified it as follows: Define the Translation' Teams Roles and Responsibilities: - Outline the distinct roles of each team member, including research team representative, translators, linguistic experts, healthcare professionals, and, if applicable, the source-language questionnaire developer |  |
|  | Clearly outline the roles of each team member, including translators, linguistic expert, healthcare professional in the team and the source-language questionnaire developer. |  |  |  |  |
| 2.4 | Assemble a Competent Team of Translators: | 4.14 (1.10) | (1) "Would change "consider using native speaker" to "Use native speaker" (2) "review of the translated material is necessary" (3) "Not sure how this item fits in (already covered in 2.1; combine with item 2.1" (4) "How is this different from 2.1; Seems a bit redundant" (5) "For me the most important point is the last which is the only mentioned as 'consider'" (5) "Isn't this the same as in point 2.1" (6) "Do we have mechanism to test the competency? or is it self reported?" (7) "Expertise not necessary, subject matter experts do not need to be the translators" (8) "Native speakers are critical" (9) "Included in item '2.1'" | Removed, and merged with item 2.1 |  |
|  | - Select translators with expertise in the subject matter of the survey. |  |  |  |  |
|  | - Ensure translators are fluent in both the source and target languages. |  |  |  |  |
|  | - Consider using native speakers for the target language to capture nuances or cultural perspectives. |  |  |  |  |
| 2.5 | Appoint Reviewers and Proofreaders: | 4.26 (0.94) | (1) "A meeting with study team and PI can be scheduled for prior to finalization" (2) "How is this different than 2.1; Seems redundant, may be merge with previous ones as this would all be part of the team work" (3) "Redundant with several previous item" | Removed, and merged with item 2.1 |  |
|  | - Designate individuals or teams to review and proofread the translated content. |  |  |  |  |
|  | - Ensure that reviewers are fluent in both languages and have a strong eye for detail. |  |  |  |  |
|  | - Encourage open communication between translators and reviewers for clarity and feedback. |  |  |  |  |
| 2.6 | Establish a Project Timeline: | 4.39 (0.77) | (1) "Testing of translated tool will help"(2) "Not sure the project/time management side of things should go into a checklist"(3) "This is just project management - could be excluded as not specific to creating a team - i.e., this is more the process. However, still useful to include." | Removed, and additional important content related to questionnaire translation process were described in the checklist guide |  |
|  | - Develop a clear timeline for the translation process, including milestones and deadlines. |  |  |  |  |
|  | - Consider buffer periods for unexpected delays or revisions. |  |  |  |  |
| 2.7 | Create a Communication Plan: | 4.48 (0.71) | (1) "Repeated review of tool with translators until satisfied" (2) "The second part of the question appears to me to be more relevant than the first" (3) "Not sure the project/time management side of things should go into a checklist. Could merge the project management items into a single item related to coordination or project management. These points are relevant for any research project, so I am unsure why they would be necessary for this specific checklist" (4) "For me the points included in this question have different levels of importance" | Removed, and additional important content related to questionnaire translation process were described in the checklist guide |  |
|  | - Set up regular communication channels for the translation team to discuss progress and address questions or concerns. |  |  |  |  |
|  | - Determine how issues and discrepancies will be resolved within the team. |  |  |  |  |
| 2.8 | rovide Necessary Resources: | 4.39 (0.77) | (1) "Solving translators issues is part of study team and PI responsibility" (2) "Ditto; see previous comments re. Project management" (3) "Same comment - difference importance levels of subitems" (4) "How will translation tools be obtained? Alos mention prefer tools that have already been validated in various languages. We may also like to define the level of quality validation that's acceptable (e.g., if a scale has been validated by 2 groups in the same language, which one to choose)" | Removed, and additional important content related to questionnaire translation process were described in the checklist guide |  |
|  | - Ensure the team has access to reference materials, glossaries, and translation tools if needed. |  |  |  |  |
|  | - Address any logistical needs, such as secure file sharing and version control. |  |  |  |  |
| 2.9 | Define Quality Assurance Procedures: | 4.70 (0.46) | (1) "Have procedures for handling discrepancies determined a priori" (2) "Could be described in manual" (3) "When do the rounds end? This may need specifying, if there can be a clear end point" | Removed, and additional important content related to questionnaire translation process were described in the checklist guide |  |
|  | - Establish a quality control process, including multiple rounds of review and feedback. |  |  |  |  |
|  | - Determine criteria for evaluating translation quality and adherence to objectives. |  |  |  |  |
| 2.10 | Ensure Transparency: | 4.73 (0.45) | (1) "Date wise" (2) "Isn't this part of the quality process" (3) "The changes ideally should be published as supplementary material of a methodology paper" | Removed, and additional important content related to questionnaire translation process were described in the checklist guide |  |
|  | - Maintain transparency in decision-making and communication within the translation team. |  |  |  |  |
|  | - Document all revisions and changes made during the translation process. |  |  |  |  |
| **Forward Translation** | |  |  |  |  |
| 3.1 | Select Translators: | 4.35 (0.91) | {1) "Material to be shared" (2) "The third point does not pertain to the selection of reviewers" (3) "Native and near-native are two different things and near-native is somehow vague" (4) "Translator should have strong background in medical terminology" (5) "May not need to be subject matter experts, these can be separate people to translators, but part of team" (6) "Redundant" | We modified and delete the last item, as follows: Select Translators: - 2+ fluent in the source language, native/near native speakers of the target language - Expertise/experience in translating in the subject matter field (if applicable) - Familiar with the culture and context of the target audience |  |
|  | - 2+ fluent in source language, native/near-native speakers of target language |  |  |  |  |
|  | - Expertise/experience in translating in the subject matter field (If applicable) |  |  |  |  |
|  | - Familiar with the culture and context of the target audience. |  |  |  |  |
|  | - Provide access to the original survey questionnaire and relevant information. |  |  |  |  |
| 3.2 | Independent Translation: | 4.48 (0.71) | (1) "Disadvantages as it takes longer time" (2) "This is another important step. Separate translation and then reconcilling is crucial to avoid possible biases of individual translaters" (3) "A sentence on auto generated translation vetting may be ideal, as that's more cost efficient and saves time" | We modified this item as follows" Independent Translation: Each translator works independently on translating the questionnaire |  |
|  | Each translator works independently on translating the questionnaire (Avoid group translations to reduce bias and ensure individual perspectives). |  |  |  |  |
| 3.3 | Identify Discrepancies: | 4.57 (0.50) | {1} "Based on available any strategy can be undertaken" (2) "2+ translators', not "two translators" (3) "Who would compare the translations and identify the discrepancies? Researchers or an independent translator?" (4) "Language proficiency of the group leaders and who takes the final call may again need specifying" | We modified this item as follows: Identify Discrepancies: - Identify who will compare the translations and identify discrepancies e.g., researchers or an independent translator - Resolve discrepancies through discussion and concensus between the translators |  |
|  | - Compare independent translations to identify discrepancies and differences. |  |  |  |  |
|  | - Resolve discrepancies through discussion and consensus between the two translators (Ensure accuracy and clarity in final forward translation). |  |  |  |  |
| 3.4 | Prioritize Understandability: | 4.48 (0.58) | (1) "Consider average reading level of target population" (2) "Common words to be used asways" (3) "While this is important, it can be quite tricky. Simplicity might some times obscurethe true nature of the original study and will need proper attention" (4) "I think some clarification around cultural context and cultural sensitivity would be helpful. This is a broad and relatively vague concept and may be interpreted differently by different people" (5) "The first item depends on target audience - part between brackets" (6) "Some of these could be separate points, e.g. cultural context can be very important and observes a point of its own and in the paper perhaps we can give specific examples" (7) "Create a glossary of key terms and concepts used in the questionnaire to ensure consistency" (8) "Maybe someone to review for cultural sensitivity? i.e.,can this be a specific role?" | Keep it to the next round |  |
|  | - Simple and clear language in the translated questionnaire (avoid medical jargon or technical terms). |  |  |  |  |
|  | - Maintain consistency in terminology throughout the questionnaire. |  |  |  |  |
|  | - Be culturally sensitive; account for conceptual differences. |  |  |  |  |
|  | - Retain original tone and intent. |  |  |  |  |
| 3.5 | Capture Conceptual Equivalents: | 4.70 (0.46) | (1) "If any such arise manual needs to state that" (2) "Ditto" (3) "I agree in concept although we should acknowledge doind so accurately relies on expertise of translators" (4) "The scale of this question has an error - there are two strongly diasgree and no strongly agree | We modified this item as follows: Capture Conceptual Equivalents: - Aim for conceptual equivalence over word-for word translation or etymological equivalence - Culturally appropriate for the audience, as certain concepts or expressions may be vary in meaning across different cultures |  |
|  | - Aim for conceptual equivalence over word-for-word translation or etymological equivalence. |  |  |  |  |
|  | - Culturally appropriate for the audience (Some concepts or expressions may have different meanings in different cultures). |  |  |  |  |
| **Back translation** | |  |  |  |  |
| 4.1 | Choose Back Translators: | 4.41 (0.94) | (1) "The procedure for translation need to be documented" (2) "The third part is not concerned about the selection of the back translators; should be move to 4.2" (3) "There is also a typo in this question (defined)" (4) "Redundant" | We modified this item as follows: Chosse Back Translators: - Well-defined criteria for translators and declare them in publication |  |
|  | - Well-defined criteria for translators and declare it in publication |  |  |  |  |
|  | - Well-defined objectives of translation and (potential) target audience |  |  | - Well-defined objectives of translation and (potential) target audience |  |
|  | - Back translators should NOT have access to the questionnaire in its original language. |  |  | - Back translators should not have access to the questionnaire in its original language. |  |
| 4.2 | Perform Independent Back Translation: | 4.52 (0.73) | (1) "Content to be emphasized" (2) "Parallelly" --> "parallel" (3) Before this item, I believe there should be another one defining the criteria for back-translators (just as it was done in the forward translation section)" (4) "Again, automated translations may be mentioned as with AI the landscape has changed, so we could consider having a separate section on that (or it could be another project for the future :))" | Keep it to the next round |  |
|  | - Parallelly comparison of forward and backward version |  |  |  |  |
|  | - Translation with lexical flexibility and readability is preferred, avoid word-by-word translation. |  |  |  |  |
| 4.3 | Implement Double-Back-Translation Methodology (optional). | 4.05 (1.13) | (1) "Scheduled meetings and reviews could be made as a protocol" (2) "I don't think this should be 'optional' " (3) "This seems like this would not be easy to do" (4) "The manager or Lead should play a major role in this step, not the third impartial translator to resolve dispute among translators" | Keep it to the next round |  |
|  | - Have two independent expert translators translate the source material into the target language. |  |  |  |  |
|  | - Use a third impartial translator to choose the better translation and resolve disputes among translators. |  |  |  |  |
|  | - Blindly back-translate the reconciled version into the source language. |  |  |  |  |
|  | - Check the back-translation for compatibility and equivalence with the source version. |  |  |  |  |
|  | - Involve multiple independent reviewers to choose the best translation for each item or suggest alternatives. |  |  |  |  |
|  | - Seek consensus among the organizing team and language coordinator. |  |  |  |  |
|  | - Test the target-language version with native-country patients and incorporate their feedback for revisions. |  |  |  |  |
| **Comparing Original and Back-Translated Versions and Reconciliation** | |  |  |  |  |
| 5.1 | Side-by-Side Comparison: | 4.68 (0.47) | (1) "Focusing on conveying the original mean accurately, maintaining the same level of formality and tone as in the original text" | Keep it to the next round |  |
|  | - Compare the original questionnaire with the back-translated version side by side. |  |  |  |  |
|  | - Identify any discrepancies or ambiguities in meaning or wording between the two versions. |  |  |  |  |
| 5.2 | Resolution of Discrepancies: | 4.73 (0.45) | (1) "Determine process for resolving discrepancies a priori" (2) "This is another crucial step in the process and helps identify culturally appropriate and sensitive issues that need to be addressed before final version is released" | Keep it to the next round |  |
|  | - Engage in discussions with the translation team to address identified discrepancies. |  |  |  |  |
|  | - Strive for consensus to create a harmonized version that retains the intended meaning. |  |  |  |  |
| 5.3 | Involvement of Translation Manager: | 4.24 (0.87) | (1) "In case of queries PI can be consulted" | Keep it to the next round |  |
|  | - The Translation Manager should lead discussions with both forward and backward translators. |  |  |  |  |
|  | - Make necessary changes to the back-translation based on input and consensus. |  |  |  |  |
|  | - Be prepared for multiple iterations if required until the translation team is satisfied with the harmonized version. |  |  |  |  |
| **Pretesting and Evaluation** | |  |  |  |  |
| 6.1 | Pretest with Target Audience: | 4.55 (0.94) | (1) "Make specific recommendations for of folks to pretest with" (2) "Pretesting with target audience often skipped but is crucial" (3) "Not needed. This item is already described below and does not seem to be appropriate as a stand alone item" | We modified this item as follows: Pretest with Target Audience: - Conduct pre testing of the translated questionnaire with members of the target audience Assess clarity, comprehension, and cultural appropriateness - Conduct cognitive interviews to uncover issues - Debrief for open-ended feedback on full experience |  |
|  | - Conduct pretesting of the translated questionnaire with members of the target audience. |  |  |  |  |
|  | - Debrief for open-ended feedback on full experience |  |  |  |  |
| 6.2 | Representative Pretest Group: | 4.67 (0.56) | (1) "Demographic diversity" --> "ensure demographic diversity" (2) "Also important to recruit people with different socio-economic and health literacy backgrounds" (3) "Yes but population subgroups based on disease are also essential (e.g., NCD is vast, thus pretest should include various disease)" (4) "And this group should be different from previous group, and also should include people of various professions, like healthy control/laymen versus diseased versus physician" | We modified this item as follows: Representative Pretest Group: - Recruit from geographic areas where the target population resides - Ensure demographic diversity by including individuals of varying ages, genders, ethnicities, and educational backgrounds in the pretesting process as appropriate. |  |
|  | - Recruit from geographic areas where target population resides. |  |  |  |  |
|  | - Demographic diversity (Include individuals of different ages, genders, ethnicities, and educational backgrounds in the pretesting process). |  |  |  |  |
| 6.3 | Consider Sample Size: | 4.41 (0.78) | (1) "As a psychometrician,I would like to add a point that the sample size required for validation (e.g., CFA, measurement invariance test) may also need to be considered" (2) "Define "uncovering issues" (3) "May need larger group if there is heterogeneous target population to ensure diversity/representation" (4) "Why 5-10? is that evidence-based?" (5) "Maybe specify this is two stages - pretesting adn the main testing?" (6) "ALWAYS calculate sample size necessary for the purpose" (7) "If they will refer to research on recommended sample sizes for cognitive interviews, why did you mention"Start with a small sample 5-10 participants for initial pretesting?" I recommend deleting the first step of this item. Also, details about the cognitive interview and what to be assessed in this step are needed" | We modified this item as follows: Consider Sample Size: - Refer to research on recommended sample sizes for cognitive interviews - Start with a small sample of participants for initial pretesting - Calculate the sample based on the estimated saturation point for uncovering issues |  |
|  | - Refer to research on recommended sample sizes for cognitive interviews |  |  |  |  |
|  | - Start with a small sample of 5-10 participants for initial pretesting. |  |  |  |  |
|  | - Calculate sample based on estimated saturation point for uncovering issues |  |  |  |  |
| 6.4 | Revisions Based on Feedback: | 4.55 (0.58) | (1) "Get feedback on instructions as well as the instrument itself" (2) "Improving --> improve" (3) "AND then retest again-this sentence may be included" (4) "From pilot testing?This may need to be clarified with regards the previous step" (5) "In the previous step, you mentioned only the qualitative assessment (i.e., cognitive interview). What about quantitative assessment?" | Keep it to the next round |  |
|  | - Incorporate necessary revisions to the questionnaire based on pretest feedback. |  |  |  |  |
|  | - Improving clarity and comprehension of questions and instructions. |  |  |  |  |
|  | - Fix any content or grammar issues raised |  |  |  |  |
| **Final Review and Proofreading** | |  |  |  |  |
| 7.1 | Expert Review: | 4.59 (0.58) | (1) "What about validation by a patient partner group? I think they need to sit/be represented in the final panel" (2) "Assess content validity, cultural, appropriateness, and clarity through expert feedback. HOW? Details are needed" | Keep it to the next round |  |
|  | - Have subject-matter experts or researchers experienced in survey methodology review the translated questionnaire. |  |  |  |  |
|  | - Assess content validity, cultural appropriateness, and clarity through expert feedback. |  |  |  |  |
| 7.2 | Final Validation: | 4.50 (0.72) | (1) "Alpha should be run on factors based on an exploratory factor analysis. Would want to pretest the survey with at least 10 people per item to run an ECA then calculate alpha" (2) "In my opinion, construct validity should also be tested via CFA. Ideally, multigroup invariance test should also be conducted to ensure that different translated versions measure the same construct in a consistent manner. If needed (when scalar invariance is not achieved), measures to address non-invariance, e.g., measurement alignment, may need to be performed" (3) "Second point: Remove "Cronbach Alpha" here - this coefficient was onl named as an example in the previous point (you could also have stated McDonald's omega or any other reliability index); likewise, feature Kappa and ICC only as examples, state instead: "measures of interrater agreement" | We modified this item as follows: Final Validation: - Assess internal consistency and reliability using appropriate indices such as Cronbach's alpha, McDonald's omega, or other relevant reliability measures - Use Cronbach's alpha to measure the internal consistency and reliability of survey items - Use measures, such as the Kappa coefficient to assess item-to-item agreement and equivalence between the source and target instruments |  |
|  | - Consider a final validation step, possibly through statistical methods like Cronbach’s alpha, to assess reliability. |  |  |  |  |
|  | - Use Cronbach’s alpha to measure the internal consistency and reliability of survey items. |  |  |  |  |
|  | - Optionally, consider Kappa coefficient and intraclass correlation coefficient to assess item-to-item agreement and equivalence between the source and target instruments. |  |  |  |  |
| 7.3 | Proofreading: | 4.73 (0.45) | None | Keep it to the next round |  |
|  | - Conduct a meticulous proofreading of the final translated questionnaire. |  |  |  |  |
|  | - Check for language accuracy, formatting consistency, and overall quality. |  |  |  |  |
|  | - Ensure that the questionnaire is free of typos, grammatical errors, and formatting inconsistencies. |  |  |  |  |
| **Post-Survey Evaluation and Continuous Improvement** | |  |  |  |  |
| 8.1 | Conduct Post-Survey Evaluations: | 4.14 (0.87) | (1) "Make sure bots can't get into your survey" (2) "Emend a verb in the first point: "Conduct a readily accessible survey" (what means "friendly" here?); last point: "provide contact information (e.g., a QR code)..." (3) "Add explicit details on how this should be done" | Keep it to the next round |  |
|  | - A friendly and readily accessible (preferred online) survey |  |  |  |  |
|  | - Feedback should be collected from both translators and readers |  |  |  |  |
|  | - Put a QR code in the survey in case participants have any feedback |  |  |  |  |
| 8.2 | Information to be collected in the post-survey Feedback from translators: | 3.77 (1.13) | (1) "Manual to be updated" (2) "First point: ??? You need to also provide item anchors - but I am not sure what is meant in this point anyway; this whole item needs clarification and improvement" (3) "Vague. For example, what is meaning of "Training Needs: Short-answer question type"? | Modify it by adding more description on the self-assessment using Likert's scale. New item as follows: Information to be collected in the post-survey feedback from translators: Self-assesment (using LIkert's scale scoring from 1 to 5). We then report the (average) overall satisfaction from the team within the questionnaire description (1) Translation Accuracy: scoring 1 (not accurate) to 5 (highly accurate) (2) Cultural Relevance: 1(not relevant) to 5 (highly relevant) (3) Linguistic Fluency: 1(not fluent) to 5 (highly fluent) (4) Contextual Adaptation: 1(not adaptable) to 5 (highly adaptable) (5) Challenges encountered: Short-answer question type (5) Training Needs: Short-answer question type feedback from readers/users (6) Suggestion for improvement: Short-answer question type feedback from readers/users using Likert's scale scoring from 1 to 5 for all questions: (A) Clarity and Understanding: Rate from 1 (totally obscure) to 5 (exceptionally clear) (B) Cultural Relevance: Evaluate from 1 (completely unrelated) to 5 (highly pertinent) (C) Linguistic Fluency: Assess from 1 (entirely non-fluent) to 5 (highly fluent) (D) Contextual Adaption: Judge from 1 (totally unsuitable) to 5 (perfectly suitable) (E) Suggestion for Improvement: Provide concise feedback |  |
|  | - Self-assessment (using Likert’s scale scoring from 1 to 5). We then report the (average) overall satisfaction from the team within the questionnaire description |  |  |  |  |
|  | - Challenges encountered: Short-answer question type |  |  |  |  |
|  |  |  |  |  |  |
| 8.3 | Perform Real-World Revisions: | 4.27 (0.81) | (1) "This question is not applicable when we disagree with the previous" (2) "Some of this last part may be redundant or less necessary" | Keep it to the next round |  |
|  | - Use the feedback from post-survey evaluations to make revisions to the translated instrument. |  |  |  |  |
|  | - Address any issues related to clarity, comprehension, or cultural sensitivity that were identified during the survey administration. |  |  |  |  |
| 8.4 | Document Revisions: | 4.41 (0.72) | "Specify what to be documented. As general feedback of this guide, explicit intsructions are needed; readers should not be expected to know the details of all items; you may need to reconsider the structure of this guide as several items seem to be redundant; a graph summarizing all steps is needed in the manuscript" | We modifiy it as follows: Document Revisions: - Maintain clear documentation of all revisions made for future reference and quality control |  |
|  | - Maintain clear documentation of all revisions made to the translated questionnaire. |  |  |  |  |
|  | - Ensure that revisions are well-documented for future reference and quality control. |  |  |  |  |

**Supplement 4: Summary of Round 2 Delphi Survey Results with Expert Panel**

| **Item** | **Item description** | **Mean (SD)** | **Expert's comments** | **Action (keep/modify/removed)** |
| --- | --- | --- | --- | --- |
| **Define Target Audience and Objectives** | | | | |
| 1.1 | Communicate the objective before the translation process, including the study objective, question objective, target respondent, and translation objective. | 4.59 (0.89) | "Communicate with whom? May be it is better to say "Specify" | Keep it to the next round |
| 1.2 | Specify the target respondent or the target population's characteristics, including:  - Nationality and regional differences  - Language dialects spoken within the community.  - Cultural background and perspectives relevant to the subject matter. | 4.23 (0.90) | (1) "Typo in item text: "target population's" - delete ' before population's" (2) "Seems a bit excessive; Some of the information listed here is hard to get" (3) "SES may be less relevant than other component" (4) "Nationality and regional difference: Differences from what? I suggest deleting this sentence "Nationality and regional differences" because it is not important; important aspects are covered in the following 4 sentences. For the socio-economic status I am not sure why this is important" (5) "Specify this objective only if really useful for the continuation of the study" | Keep it to the next round |
|  | - Educational levels of the target population - Socioeconomic status |  |  |  |
| 1.3 | Evaluate the importance of conceptual translation (instead of verbatim translation) to enhance the overall quality of the translated questionnaire. | 4.27 (1.09) | (1) "If required by the translator conceptual framework can be shared" (2) "How would you evaluate the importance of conceptual translation? Not sure I understand this" (3) "This is already known as a core principle of any translation process" | item removed |
| 1.4 | Engage with a diverse group of stakeholders, such as interviewers, pretest respondents, and experts in the field, to collect input and perspectives. | 4.27 (0.96) | "I would also include members of the target audience in the earliest phases" | Keep it to the next round |
| **Create a Translation Team** | |  |  |  |
| 2.1 | Form a translation team, consist of:  - Translation Lead or Manager  - At last two proficient translator, preferably native speakers of the target language, and, if applicable, | 4.27 (0.86) | (1) "Agree but incomplete - back translation are essential and missing" (2) Rather than a native speaker I would go with a person living in the country with the targeted language for 5 years or more" | Keep it to the next round |
|  | - The source-language Questionnaire Developer. |  |  |  |
| 2.2 | Appoint a qualified Translation Manager:  - Identify an experienced individual who will oversee the entire translation process (manage resources, direct and coordinate procedural steps, documentation of results and facilitate discussion). | 4.18 (0.94) | (1) "Typo: 'survey's (2) "how to verify?" | Keep it to the next round |
|  | - Verify that the Translation Manager has a strong understanding of the survey's objectives and the target audience. |  |  |  |
| 2.3 | Define the Translation Team's Roles and Responsibilities: | 4.23 (0.85) | "Typo: Team's - this kind of err repeteadly appeared up to ere. Please check all furter items as well" | Keep it to the next round |
|  | Outline the distinct roles of each team representative, translators, linguistic experts, healthcare professionals, and, if applicable, the source-language questionnaire developer. |  |  |  |
| **Forward Translation** | |  |  |  |
| 3.1 | Select Translators:  - 2+ fluent in source language, native/near-native speakers of target language  - Expertise/experience in translating in the subject matter field (If applicable) | 4.36 (0.88) | "I have commented in previous version - there is a mistake in the response options of this question" | Keep it to the next round |
|  | - Familiar with the culture and context of the target audience. |  |  |  |
| 3.2 | Independent Translation: | 4.45 (0.72) | "same here" | Keep it to the next round |
|  | Each translator works independently on translating the questionnaire |  |  |  |
| 3.3 | Identify Discrepancies:  - Identify who will compare the translations and Identify discrepancies e.g., researchers or an independent translators | 4.41 (0.98) | (1) "Insert a comma before the "e.g.," (2) "same" | Keep it to the next round |
|  | - Resolve discrepancies through discussion and consensus between the translators |  |  |  |
| 3.4 | Prioritize Understandability:  - Simple and clear language in the translated questionnaire (avoid medical jargon or technical terms).  - Maintain consistency in terminology throughout the questionnaire.  - Be culturally sensitive; account for conceptual differences. | 4.41 (0.89) | (1) "same" (2) "Add "Use" to the first sentence" | Modify by adding "Use" to the first sentence as suggested |
|  | - Retain original tone and intent. |  |  |  |
| 3.5 | Capture Conceptual Equivalents:  - Aim for conceptual equivalence over word-for-word translation or etymological equivalence. | 4.48 (0.91) | (1) "first point may be a repetition" (2) "The second sentence "Culturaly appropriate for the audience, as…" is not necessary as it is already included in the previous sentence" | Modify by deleted "..as…" as suggested by the expert |
|  | - Culturally appropriate for the audience as certain concepts or expressions may have different meanings across different cultures. |  |  |  |
| **Back translation** | |  |  |  |
| 4.1 | Choose Back Translators:  - Well-defined criteria for translators and declare it in publication  - Well-defined objectives of translation and (potential) target audience | 4.36 (0.93) | (1) "Formulate well-defined criteria… and report them in the publication", "formulate well-defined objectives…" (2) "Incomplete - does not mention number, fluency or independent working" | Modified the item as follows: Choose Back Translators: - Formulate well-defined criteria for translators and report them in publication - Formulate well-defined objectives of translation and (potential) target audience - Two independent back translators is recommended to minimize risk of bias. Any conflict should be addressed by agreement or by a third (senior) reviewer. - Back translators should not have access to the questionnaire in its original language. |
|  | - Back translators should NOT have access to the questionnaire in its original language. |  |  |  |
| 4.2 | Perform Independent Back Translation:  - Parallelly comparison of forward and backward version | 4.45 (0.89) | "Parallel comparison…" | Modified the item as follows: Perform Independent Back Translation: - Parallel comparison of forward and backward version-  Prefer flexible and readable translation over word-for-word. |
|  | - Translation with lexical flexibility and readability is preferred, avoid word-by-word translation. |  |  |  |
| 4.3 | Implement Double-Back-Translation Methodology (optional).  - Have two independent expert translators translate the source material into the target language.  - Use a third impartial translator to choose the better translation and resolve disputes among translators.  - Blindly back-translate the reconciled version into the source language.  - Check the back-translation for compatibility and equivalence with the source version.  - Involve multiple independent reviewers to choose the best translation for each item or suggest alternatives.  - Seek consensus among the organizing team and language coordinator. | 3.71 (1.20) | (1) "Most research teams do not have this level of support or resources at their disposal" (2) "For me this is not optional but a must have" (3) "Agree on blinding, disagree on selecting one prefered solution" | Modified the item as follows: Implement Double-Back-Translation Methodology (optional). - Have two independent expert translators translate the source material into the target language. - Involve a third impartial translator to resolve disputes between two translators and consolidate a final version. - Blindly back-translate the reconciled version into the source language. - Check the back-translation for compatibility and equivalence with the source version. - Involve multiple independent reviewers to choose the best translation for each item or suggest alternatives. - Seek consensus among the organizing team and language coordinator. - Test the target-language version with native-country patients and incorporate their feedback for revisions. |
|  | - Test the target-language version with native-country patients and incorporate their feedback for revisions. |  |  |  |
| **Comparing Original and Back-Translated Versions and Reconciliation** | |  |  |  |
| 5.1 | Side-by-Side Comparison:  - Compare the original questionnaire with the back-translated version side by side. | 4.32 (0.97) | "Could note the discrepancies may occur given tarnslating for concept, not word fo word" | Keep it to the next round |
|  | - Identify any discrepancies or ambiguities in meaning or wording between the two versions. |  |  |  |
| 5.2 | Resolution of Discrepancies:  - Engage in discussions with the translation team to address identified discrepancies. | 4.41 (0.89) | (1) "depending on the level of inconsistency, restarting the process may be needed" (2) "Opportunity also to improve original version" | Modified this item as follows: Resolution of Discrepancies: - Engage in discussions with the translation team to address identified discrepancies. - Strive for consensus to create a harmonized version that retains the original meaning. - High level of inconsistency may result in restarting the process from step 3 (Forward translation) |
|  | - Strive for consensus to create a harmonized version that retains the intended meaning. |  |  |  |
| 5.3 | Involvement of Translation Manager:  - The Translation Manager should lead discussions with both forward and backward translators.  - Make necessary changes to the back-translation based on input and consensus. | 4.09 (0.85) | (1) "Either drop "The translation manager i the first point or amend in points 2 and 3" (2) "Could note the discrepancies may occur given tarnslating for concept, not word fo word" | Keep it to the next round |
|  | - Be prepared for multiple iterations if required until the translation team is satisfied with the harmonized version. |  |  |  |
| **Pretesting and Evaluation** | |  |  |  |
| 6.1 | Pretest with Target Audience:  - Conduct pretesting of the translated questionnaire with members of the target audience. - Asses clarity, comprehension, and culural appropriateness | 4.45 (0.94) | "What is the difference between the 2nd and the 3rd points?" | Keep it to the next round |
|  | - Conduct cognitive interviews to uncover issues - Debrief for open-ended feedback on full experience |  |  |  |
| 6.2 | Representative Pretest Group:  - Recruit from geographic areas where target population resides. | 4.32 (0.92) | None | Keep it to the next round |
|  | - Ensure demographic diversity by including individuals of varying ages, genders, ethnicities, and educational backgrounds in the pretesting process as approriate. |  |  |  |
| 6.3 | Consider Sample Size:  - Refer to research on recommended sample sizes for cognitive interviews  - Start with a small sample of participants for initial pretesting. | 4.45 (0.78) | "Calcualte the sample based on the estimated saturation point for uncovering issues: Saturation is for qualitative approaches, and there is no sample size cakculation for qualitative approaches. Maybe I misunderstood what you want to say here, Maybe you need to specify the sample size here because readers should not need to refer to another reference to know this information. | Keep it to the next round |
|  | - Calculate sample based on estimated saturation point for uncovering issues |  |  |  |
| 6.4 | Revisions Based on Feedback:  - Incorporate necessary revisions to the questionnaire based on pretest feedback.  - Improving clarity and comprehension of questions and instructions. | 4.55 (0.89) | None | Keep it to the next round |
|  | - Fix any content or grammar issues raised |  |  |  |
| **Final Review and Proofreading** | |  |  |  |
| 7.1 | Expert Review:  - Have subject-matter experts or researchers experienced in survey methodology review the translated questionnaire. | 4.36 (0.93) | (1) "Should this come earlier in the process" (2) "Typo in response option" (3) "For the 2nd point, explain how" (4) "Most important will check the mode of administration; digital interface might require some compromise" | Keep it to the next round |
|  | - Assess content validity, cultural appropriateness, and clarity through expert feedback. |  |  |  |
| 7.2 | Final Validation:  - Assess internal consistency and reliability using appropriate indices such as Cronbach's alpha, McDonald's omega, or other relevant reliability measures  - Use Cronbach’s alpha to measure the internal consistency and reliability of survey items. | 4.32 (1.26) | (1) "Points 1 and 2 are redundant; I'd keep point 1, because it names also alternatives to Cronbach alpha; I'm not sure, how Kappa or ICCs could be applied here - this needs more explanation" (2) "There are step missing between 7.1 and 7,2. You cannot undertake 7.2 with a small sample applied only once" (3) Delete the 2nd and 3rd points and include their content in the first point. You did not include measures of validity !!" (4) "Will do that in clinical trials not as a stand alone/ small sample though" | Keep it to the next round |
|  | - Use measures such as the Kappa coefficient and intraclass correlation coefficient to assess item-to-item agreement and equivalence between the source and target instruments. |  |  |  |
| 7.3 | Proofreading:  - Conduct a meticulous proofreading of the final translated questionnaire.  - Check for language accuracy, formatting consistency, and overall quality. | 4.5 (0.94) | (1) "Should this come before final validation study?" (2) "Assessing appropriate spaces used for each item in questionnaire/tool" (3) "Will still recommend proofreading on final format, such as phone" | Keep it to the next round |
|  | - Ensure questionnaire is free of typos, grammatical errors, and formatting inconsistencies. |  |  |  |
| **Post-Survey Evaluation and Continuous Improvement** | |  |  |  |
| 8.1 | Conduct Post-Survey Evaluations:  - A friendly and readily accessible (preferred online) survey  - Feedback should be collected from both translators and readers | 4.00 (1.09) | "QR is not a must; other methods are also OK." | Modify the item as follows: Conduct Post-Survey Evaluations: - A conventional and readily accessible (preferred online) survey - Feedback should be collected from both translators and readers - (Optional) Put a QR code in the survey in case participants have any feedback |
|  | - Put a QR code in the survey in case participants have any feedback |  |  |  |
| 8.2 | Information to be collected in the post-survey Feedback from translators:  - Self-assessment (using Likert’s scale scoring from 1 to 5). We then report the (average) overall satisfaction from the team within the questionnaire description | 3.9 (0.99) | (1) "Likert scale"; avoid use of "we" or other pronouns; also, this item is very long - use a number of items instead?" (2) "This is perfect as you explain in detail. You should also describe other item in detail." | Item removed |
|  | (1) Translation Accuracy: scoring 1 (not accurate) to 5 (highly accurate) (2) Cultural Relevance: 1(not relevant) to 5 (highly relevant) (3) Linguistic Fluency: 1(not fluent) to 5 (highly fluent) (4) Contextual Adaptation: 1(not adaptable) to 5 (highly adaptable) (5) Challenges encountered: Short-answer question type (5) Training Needs: Short-answer question type feedback from readers/users (6) Suggestion for improvement: Short-answer question type feedback from readers/users using Likert's scale scoring from 1 to 5 for all questions: (A) Clarity and Understanding: Rate from 1 (totally obscure) to 5 (exceptionally clear) (B) Cultural Relevance: Evaluate from 1 (completely unrelated) to 5 (highly pertinent) (C) Linguistic Fluency: Assess from 1 (entirely non-fluent) to 5 (highly fluent) (D) Contextual Adaption: Judge from 1 (totally unsuitable) to 5 (perfectly suitable) (E) Suggestion for Improvement: Provide concise feedback |  |  |  |
| 8.3 | Perform Real-World Revisions:  - Use the feedback from post-survey evaluations to make revisions to the translated instrument. | 4.05 (1.09) | (1) "from the post-survey..." (2) "Should be done already in pretesting phase" | Keep it to the next round (become item 8.2) |
|  | - Address any issues related to clarity, comprehension, or cultural sensitivity that were identified during the survey administration. |  |  |  |
| 8.4 | Document Revisions: | 4.23 (1.04) | None | Keep it to the next round (becomeitem 8.3) |
|  | - Maintain clear documentation of all revisions made for future reference and quality control |  |  |  |
